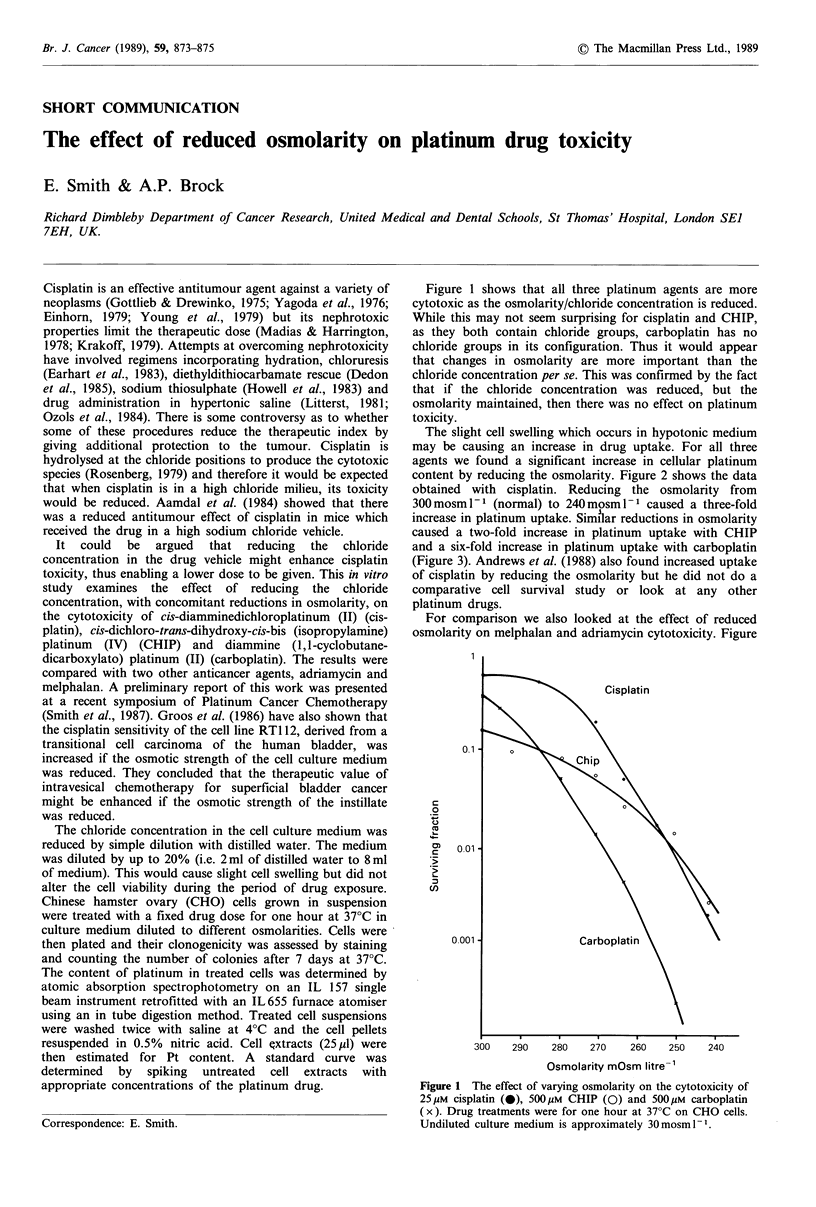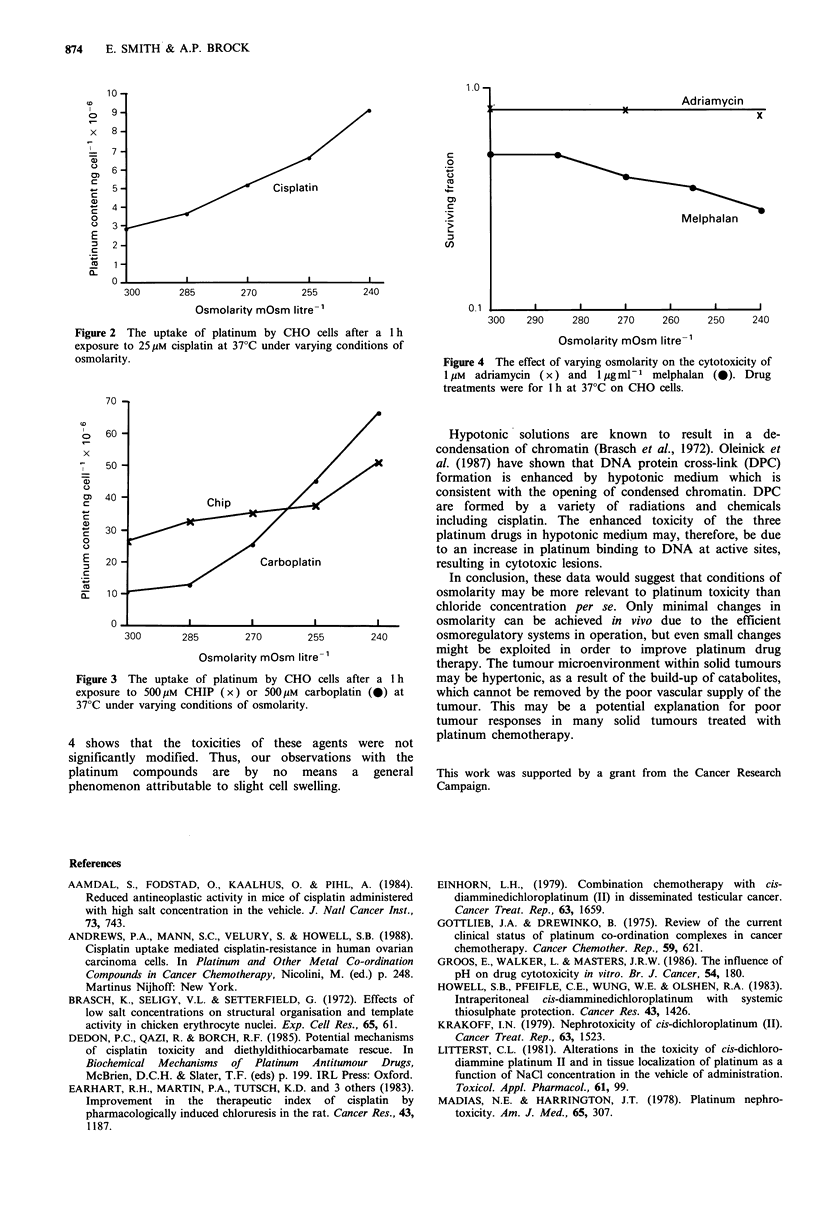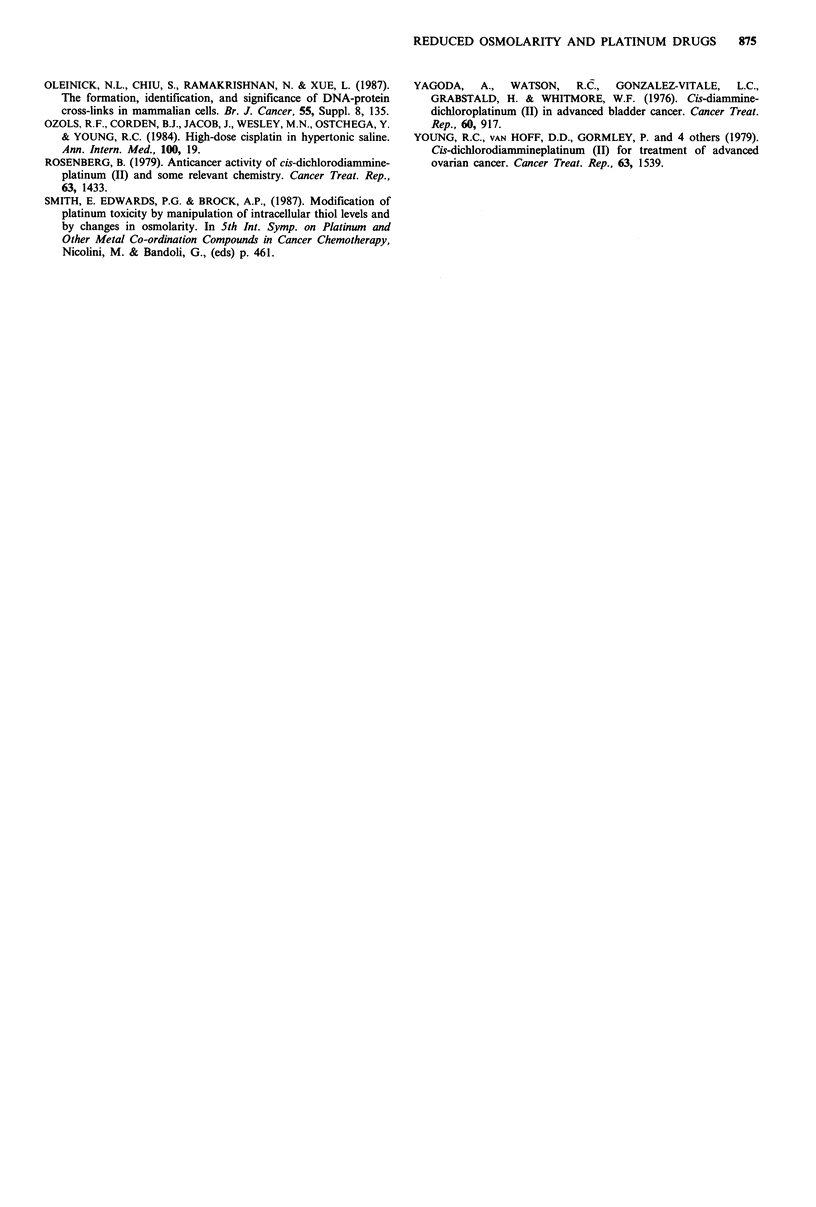# The effect of reduced osmolarity on platinum drug toxicity.

**DOI:** 10.1038/bjc.1989.185

**Published:** 1989-06

**Authors:** E. Smith, A. P. Brock

**Affiliations:** Richard Dimbleby Department of Cancer Research, United Medical School, St Thomas' Hospital, London, UK.


*Br. J. Cancer* (1989), **59**, 873–875 © The Macmillan Press Ltd., 1989

SHORT COMMUNICATION

### The effect of reduced osmolarity on platinum drug toxicity

E. Smith & A.P. Brock

*Richard Dimbleby Department of Cancer Research, United Medical and Dental Schools, St Thomas' Hospital, London SE1 7EH, UK.*

Cisplatin is an effective antitumour agent against a variety of neoplasms (Gottlieb & Drewinko, 1975; Yagoda *et al.*, 1976; Einhorn, 1979; Young *et al.*, 1979) but its nephrotoxic properties limit the therapeutic dose (Madias & Harrington, 1978; Krakoff, 1979). Attempts at overcoming nephrotoxicity have involved regimens incorporating hydration, chloruresis (Earhart *et al.*, 1983), diethyldithiocarbamate rescue (Dedon *et al.*, 1985), sodium thiosulphate (Howell *et al.*, 1983) and drug administration in hypertonic saline (Litterst, 1981; Ozols *et al.*, 1984). There is some controversy as to whether some of these procedures reduce the therapeutic index by giving additional protection to the tumour. Cisplatin is hydrolysed at the chloride positions to produce the cytotoxic species (Rosenberg, 1979) and therefore it would be expected that when cisplatin is in a high chloride milieu, its toxicity would be reduced. Aamdal *et al.* (1984) showed that there was a reduced antitumour effect of cisplatin in mice which received the drug in a high sodium chloride vehicle.

It could be argued that reducing the chloride concentration in the drug vehicle might enhance cisplatin toxicity, thus enabling a lower dose to be given. This *in vitro* study examines the effect of reducing the chloride concentration, with concomitant reductions in osmolarity, on the cytotoxicity of *cis*-diamminedichloroplatinum (II) (cisplatin), *cis*-dichloro-*trans*-dihydroxy-*cis*-bis (isopropylamine) platinum (IV) (CHIP) and diammine (1,1-cyclobutanedicarboxylato) platinum (II) (carboplatin). The results were compared with two other anticancer agents, adriamycin and melphalan. A preliminary report of this work was presented at a recent symposium of Platinum Cancer Chemotherapy (Smith *et al.*, 1987). Groos *et al.* (1986) have also shown that the cisplatin sensitivity of the cell line RT112, derived from a transitional cell carcinoma of the human bladder, was increased if the osmotic strength of the cell culture medium was reduced. They concluded that the therapeutic value of intravesical chemotherapy for superficial bladder cancer might be enhanced if the osmotic strength of the instillate was reduced.

The chloride concentration in the cell culture medium was reduced by simple dilution with distilled water. The medium was diluted by up to 20% (i.e. 2 ml of distilled water to 8 ml of medium). This would cause slight cell swelling but did not alter the cell viability during the period of drug exposure. Chinese hamster ovary (CHO) cells grown in suspension were treated with a fixed drug dose for one hour at 37°C in culture medium diluted to different osmolarities. Cells were then plated and their clonogenicity was assessed by staining and counting the number of colonies after 7 days at 37°C. The content of platinum in treated cells was determined by atomic absorption spectrophotometry on an IL 157 single beam instrument retrofitted with an IL655 furnace atomiser using an in tube digestion method. Treated cell suspensions were washed twice with saline at 4°C and the cell pellets resuspended in 0.5% nitric acid. Cell extracts (25 $\mu$l) were then estimated for Pt content. A standard curve was determined by spiking untreated cell extracts with appropriate concentrations of the platinum drug.

Correspondence: E. Smith.

Figure 1 shows that all three platinum agents are more cytotoxic as the osmolarity/chloride concentration is reduced. While this may not seem surprising for cisplatin and CHIP, as they both contain chloride groups, carboplatin has no chloride groups in its configuration. Thus it would appear that changes in osmolarity are more important than the chloride concentration *per se*. This was confirmed by the fact that if the chloride concentration was reduced, but the osmolarity maintained, then there was no effect on platinum toxicity.

The slight cell swelling which occurs in hypotonic medium may be causing an increase in drug uptake. For all three agents we found a significant increase in cellular platinum content by reducing the osmolarity. Figure 2 shows the data obtained with cisplatin. Reducing the osmolarity from 300 mosm $l^{-1}$ (normal) to 240 mosm $l^{-1}$ caused a three-fold increase in platinum uptake. Similar reductions in osmolarity caused a two-fold increase in platinum uptake with CHIP and a six-fold increase in platinum uptake with carboplatin (Figure 3). Andrews *et al.* (1988) also found increased uptake of cisplatin by reducing the osmolarity but he did not do a comparative cell survival study or look at any other platinum drugs.

For comparison we also looked at the effect of reduced osmolarity on melphalan and adriamycin cytotoxicity. Figure

**Figure 1** The effect of varying osmolarity on the cytotoxicity of 25 $\mu$M cisplatin (●), 500 $\mu$M CHIP (○) and 500 $\mu$M carboplatin (×). Drug treatments were for one hour at 37°C on CHO cells. Undiluted culture medium is approximately 30 mosm $l^{-1}$.

**Figure 2** The uptake of platinum by CHO cells after a 1 h exposure to 25 $\mu$M cisplatin at 37°C under varying conditions of osmolarity.

**Figure 3** The uptake of platinum by CHO cells after a 1 h exposure to 500 $\mu$M CHIP (×) or 500 $\mu$M carboplatin (●) at 37°C under varying conditions of osmolarity.

4 shows that the toxicities of these agents were not significantly modified. Thus, our observations with the platinum compounds are by no means a general phenomenon attributable to slight cell swelling.

**Figure 4** The effect of varying osmolarity on the cytotoxicity of 1 $\mu$M adriamycin (×) and 1 $\mu$g ml$^{-1}$ melphalan (●). Drug treatments were for 1 h at 37°C on CHO cells.

Hypotonic solutions are known to result in a decondensation of chromatin (Brasch *et al.*, 1972). Oleinick *et al.* (1987) have shown that DNA protein cross-link (DPC) formation is enhanced by hypotonic medium which is consistent with the opening of condensed chromatin. DPC are formed by a variety of radiations and chemicals including cisplatin. The enhanced toxicity of the three platinum drugs in hypotonic medium may, therefore, be due to an increase in platinum binding to DNA at active sites, resulting in cytotoxic lesions.

In conclusion, these data would suggest that conditions of osmolarity may be more relevant to platinum toxicity than chloride concentration *per se*. Only minimal changes in osmolarity can be achieved *in vivo* due to the efficient osmoregulatory systems in operation, but even small changes might be exploited in order to improve platinum drug therapy. The tumour microenvironment within solid tumours may be hypertonic, as a result of the build-up of catabolites, which cannot be removed by the poor vascular supply of the tumour. This may be a potential explanation for poor tumour responses in many solid tumours treated with platinum chemotherapy.

This work was supported by a grant from the Cancer Research Campaign.

### References

AAMDAL, S., FODSTAD, O., KAALHUS, O. & PIHL, A. (1984). Reduced antineoplastic activity in mice of cisplatin administered with high salt concentration in the vehicle. *J. Natl Cancer Inst.*, **73**, 743.

ANDREWS, P.A., MANN, S.C., VELURY, S. & HOWELL, S.B. (1988). Cisplatin uptake mediated cisplatin-resistance in human ovarian carcinoma cells. In *Platinum and Other Metal Co-ordination Compounds in Cancer Chemotherapy*, Nicolini, M. (ed.) p. 248. Martinus Nijhoff: New York.

BRASCH, K., SELIGY, V.L. & SETTERFIELD, G. (1972). Effects of low salt concentrations on structural organisation and template activity in chicken erythrocyte nuclei. *Exp. Cell Res.*, **65**, 61.

DEDON, P.C., QAZI, R. & BORCH, R.F. (1985). Potential mechanisms of cisplatin toxicity and diethyldithiocarbamate rescue. In *Biochemical Mechanisms of Platinum Antitumour Drugs*, McBrien, D.C.H. & Slater, T.F. (eds) p. 199. IRL Press: Oxford.

EARHART, R.H., MARTIN, P.A., TUTSCH, K.D. and 3 others (1983). Improvement in the therapeutic index of cisplatin by pharmacologically induced chloruresis in the rat. *Cancer Res.*, **43**, 1187.

EINHORN, L.H., (1979). Combination chemotherapy with *cis*-diamminedichloroplatinum (II) in disseminated testicular cancer. *Cancer Treat. Rep.*, **63**, 1659.

GOTTLIEB, J.A. & DREWINKO, B. (1975). Review of the current clinical status of platinum co-ordination complexes in cancer chemotherapy. *Cancer Chemother. Rep.*, **59**, 621.

GROOS, E., WALKER, L. & MASTERS, J.R.W. (1986). The influence of pH on drug cytotoxicity *in vitro*. *Br. J. Cancer*, **54**, 180.

HOWELL, S.B., PFEIFLE, C.E., WUNG, W.E. & OLSHEN, R.A. (1983). Intraperitoneal *cis*-diamminedichloroplatinum with systemic thiosulphate protection. *Cancer Res.* **43**, 1426.

KRAKOFF, I.N. (1979). Nephrotoxicity of *cis*-dichloroplatinum (II). *Cancer Treat. Rep.*, **63**, 1523.

LITTERST, C.L. (1981). Alterations in the toxicity of *cis*-dichlorodiammine platinum II and in tissue localization of platinum as a function of NaCl concentration in the vehicle of administration. *Toxicol. Appl. Pharmacol.*, **61**, 99.

MADIAS, N.E. & HARRINGTON, J.T. (1978). Platinum nephrotoxicity. *Am. J. Med.*, **65**, 307.

OLEINICK, N.L., CHIU, S., RAMAKRISHNAN, N. & XUE, L. (1987). The formation, identification, and significance of DNA-protein cross-links in mammalian cells. *Br. J. Cancer*, **55**, Suppl. 8, 135.

OZOLS, R.F., CORDEN, B.J., JACOB, J., WESLEY, M.N., OSTCHEGA, Y. & YOUNG, R.C. (1984). High-dose cisplatin in hypertonic saline. *Ann. Intern. Med.*, **100**, 19.

ROSENBERG, B. (1979). Anticancer activity of *cis*-dichlorodiammine-platinum (II) and some relevant chemistry. *Cancer Treat. Rep.*, **63**, 1433.

SMITH, E. EDWARDS, P.G. & BROCK, A.P., (1987). Modification of platinum toxicity by manipulation of intracellular thiol levels and by changes in osmolarity. In *5th Int. Symp. on Platinum and Other Metal Co-ordination Compounds in Cancer Chemotherapy*, Nicolini, M. & Bandoli, G., (eds) p. 461.

YAGODA, A., WATSON, R.C., GONZALEZ-VITALE, L.C., GRABSTALD, H. & WHITMORE, W.F. (1976). *Cis*-diammine-dichloroplatinum (II) in advanced bladder cancer. *Cancer Treat. Rep.*, **60**, 917.

YOUNG, R.C., VAN HOFF, D.D., GORMLEY, P. and 4 others (1979). *Cis*-dichlorodiammineplatinum (II) for treatment of advanced ovarian cancer. *Cancer Treat. Rep.*, **63**, 1539.